# Assessment of Maxillary Molars Interradicular Septum Morphological Characteristics as Criteria for Ideal Immediate Implant Placement—The Advantages of Cone Beam Computed Tomography Analysis

**DOI:** 10.3390/diagnostics12041010

**Published:** 2022-04-16

**Authors:** Zlata Rajkovic Pavlovic, Pavle Milanovic, Milica Vasiljevic, Nemanja Jovicic, Aleksandra Arnaut, Djurdjina Colic, Marijana Petrovic, Momir Stevanovic, Dragica Selakovic, Gvozden Rosic

**Affiliations:** 1 Department of Dentistry, Faculty of Medical Sciences, University of Kragujevac, 34000 Kragujevac, Serbia; zlatakg@yahoo.com (Z.R.P.); milicavaska13@gmail.com (M.V.); sandra11_92@yahoo.com (A.A.); djurdjinacolic@gmail.com (D.C.); m.petrovicstom@gmail.com (M.P.); momirstevanovic7@gmail.com (M.S.); 2 Department of Histology and Embryology, Faculty of Medical Sciences, University of Kragujevac, 34000 Kragujevac, Serbia; nemanjajovicic.kg@gmail.com; 3 Department of Physiology, Faculty of Medical Sciences, University of Kragujevac, 34000 Kragujevac, Serbia; grosic@medf.kg.ac.rs

**Keywords:** maxillary molars interradicular septum, cone beam computed tomography—CBCT, morphometric analysis, immediate implant placement

## Abstract

The aim of this study was to evaluate the interradicular septum bone morphometric characteristics using cone beam computed tomography (CBCT) images, as well as to establish quantitative shortcuts to allow clinicians to make a faster and more reliable plan for immediate implant placement in the maxillary molars area. This retrospective quantitative study was conducted on CBCT images obtained from 100 patients. The morphometric analysis of the maxillary molars region was based on the parameters obtained on the sagittal and axial slices. The analysis performed on sagittal slices showed that the first maxillary molars had a wider interradicular septum when compared to the second molars, but the septum height in the first molars was significantly below the height in the second maxillary molars. The axial CBCT slices analysis showed that both interradicular septum perimeter and surface area were significantly more pronounced in the first than in the second maxillary molars. The interradicular furcation angle significantly correlated with the surface area (positively) and septum height (negatively) for both molars. The results of this study may recommend CBCT image analysis as a useful tool in predefining the circumstances that can allow for substantially better planning of immediate implant placement procedures in the region of maxillary molars.

## 1. Introduction

The interradicular septum is the bone structure located between dental sockets that separates the tooth roots from furcation line to the apical limit of the roots [1]. The upper molars are usually multi-rooted teeth, predominantly with three roots, so the septal bone between them usually expresses triangular shapes [2]. Although the interradicular bone septum represents the ideal place for immediate implant placement in the posterior region [3], in some cases, insufficient interradicular bone septum dimensions could compromise implant placement procedures [4]. In order to achieve more successful implant therapy using the interradicular bone septum as the implant place, Agostinelli and coworkers [5] reported the importance of morphological characteristics of interradicular bone septum as the criteria determining the therapy.

It is well known that a significant improvement in the analysis of this maxillary region was achieved by using cone beam computed tomography (CBCT). With all respect to the most commonly used radiographic diagnostic procedures in dentistry, the use of CBCT has overcome the shortcomings of other radiological methods such as the superimpositions and distortions of 2D radiography, limited access of computed tomography, poor resolution, difficulties in interpretation in dentistry, longer scanning time, high cost, disturbance of metal artifacts, and high radiation exposure of computed tomography and multi-sliced computed tomography [6,7]. Additionally, the advantages of CBCT have reduced radiation by collimating the primary X-ray beam to the area of interest [8,9]; the submillimeter resolution accuracy of the image is precise enough for implant planning and orthodontic analysis [10,11,12], a rapid scan time has reduced the possibility of artifact making by the patient moving [8], and display modes unique to maxillofacial imaging sets by a series of segmented non-orthogonally planes can also provide oblique, curved planar reformation distortion-free images, serial cross-sectional reformation, and real size data [6,8].

The use of CBCT methodology has already proven remarkable characteristics for the subtle morphometric analysis of structures that may be of potential interest in planning the procedures accompanied by implant placement [13,14,15].

In addition to morphological examination of anatomical structures, CBCT and related software also have the possibility for the insertion of the virtual implant [16,17]. Using a virtual implant assessment program, Chen and collaborators [18] estimated the interradicular septum bone in the region of the first and second mandibular molars, as a place for implant placement, since the insufficient dimension of the interradicular septum could present a gap between the implant and bone [3,19]. Moreover, those circumstances had been reported to result in non-osseointegrated or implant failure [20]. In order to achieve a better insight into this issue, and to allow for the potential guideline for clinicians, Smith and coworkers classified the socket type by implant position into the interradicular septum [19]. According to their classification, the A-type socket represents a sufficient space for a stable implant, completely surrounded by septal bone, with no gap. The B-type socket represents the stable implant, but with prolapse from septum that perforates into the socket, so there is a gap between the implant and the wall of alveoli. Finally, the C-type socket is defined as alveoli without interradicular septum that requires a wider implant to achieve the primary fixation [19]. On the other hand, in the study presented by Bleyan and colleagues [21], the initial interradicular septum width proposed as the favorable septum width was over 2 mm. According to their classification (SI width >4 mm, SII width >3–4 mm, SIII width >2–3 mm), the septum with a width of 2 mm or less should not be assessable for immediate implantation [21].

The literature data offer a variety of studies (sometimes contradictory) considering the interradicular septum dimensions related to the final outcome of the prime implant fixation. Thus, it has been reported that minimal bone height should be predefined at 5 mm [22], but also at 10 mm [23]; while the critical value for the septum width was set at 3 mm [24] or more than 2.5 mm [21]. Interestingly, it has also been reported that there are significant differences in the interradicular septum morphometric characteristics between the first and second maxillary molars. However, the diversity of findings was obvious again, since Agustineli and coworkers [5] reported that the interradicular septum in the area of the first upper molars was higher in comparison to the second molars, while Choi and colleagues [25] showed the opposite results.

Taken together, it is obvious that the morphometric analysis of the maxillary molars region using the advanced diagnostic methodology has potential clinical importance. Therefore, the aim of this study was to evaluate the interradicular septum bone morphometric characteristics using CBCT images, in order to estimate the previous findings, as well as to establish quantitative shortcuts that can be useful for clinicians to make a faster and more reliable plan for immediate implant placement in maxillary molars area.

## 2. Materials and Methods

### 2.1. Study Design

This retrospective quantitative study was conducted on CBCT images obtained from patients at the Department of Dentistry of the Faculty of Medical Sciences, University of Kragujevac, Serbia, during January and February 2022, and carried out in accordance with the Declaration of Helsinki and with the approval by the institutional review board of Faculty of Medical Sciences, University of Kragujevac (approval ID 01-14727). The inclusion criteria for involvement in this study were as follows: patients over 18 years of age, at least one maxillary molar present, and formal consent for the usage of personal clinical data for scientific purposes. The excluding criteria were predefined as low image quality and no impacted teeth or other pathology present in the area of interest. According to those criteria, the total number of participants included in this study was 100, with the total number of individual maxillary molar images of 213.

The images were obtained using an Orthophos XG 3D device (Sirona Dental Systems GmbH, Bensheim, Germany), and analyzed using GALAXIS software v1.9.4 (Sirona Dental Systems GmbH, Bensheim, Germany) according to the previously described procedure [13].

**Figure 1:**
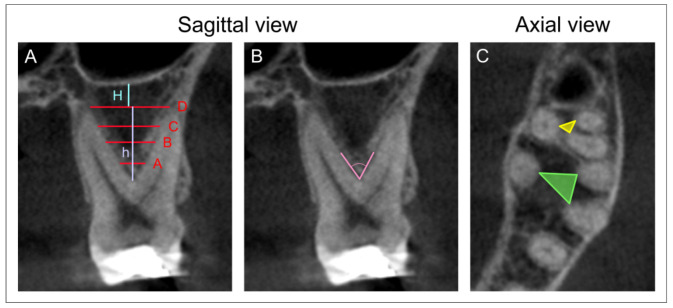
CBCT scans and landmarks of interest. (**A**)—Sagittal view: the interradicular septum width at level A (at 2 mm distance from furcation), B (at the mid length of h), C (at 2 mm distance from the interradicular septum base), and D (at the interradicular septum base); the interradicular septum height—h; the distance between the interradicular septum base and the sinus floor—H. (**B**)—Sagittal view: the interradicular furcation angle. (**C**)—Axial view: the interradicular septum surface perimeter and the surface area (for the first molars—green, and the second molars—yellow line).

The morphometric analysis of the maxillary molars region was based on the parameters obtained on the sagittal and axial slices. The sagittal views were used for measurement of the following parameters: the interradicular septum width at different levels (Figure 1A, in mm), the interradicular septum height (Figure 1A, in mm), the interradicular furcation angle (Figure 1B, in degree), and the distance between the interradicular septum base and the sinus floor (Figure 1A, in mm). The axial images (Figure 1C) were used for quantification of the interradicular septum surface perimeter according to the previously described methodology [5], followed by the surface area calculation according to Heron’s formula [26,27,28]. All parameters were analyzed by two independent experimenters who were blind to the protocol and showed high inter-rater reliability (Pearsons’s r = 0.95). The mean value for each parameter was taken for further evaluation.

**Figure 2:**
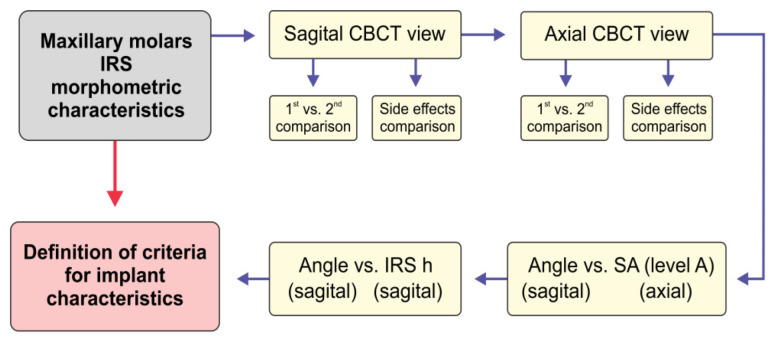
The scheme for the analysis of maxillary molars’ interradicular septum morphological characteristics as criteria for ideal immediate implant placement. The interradicular septum (IRS) parameters were used for the comparison between the first (1st) and second (2nd) and between the left and right (side effect) maxillary molars at different cross-sections of the sagittal and axial view. The interradicular furcation angle was then tested for the interconnection with the surface area (SA) at the level A, and IRS height (h).

The analysis of the obtained parameters was performed according to the following algorithm (Figure 2):

### 2.2. Statistical Analysis

All data obtained in this study were expressed as means ± SEM. Following initial submission to Levene’s test for homogeneity of variance and to the Shapiro–Wilk test of normality, the comparisons between groups were performed using the Student’s t-test. Furthermore, Pearson’s coefficient of correlation was used to analyze relationships between parameters, and simple linear regression analyses were performed. A value of *p* < 0.05 was considered to be significant. Statistical analysis was performed with the SPSS version 20.0 statistical package (IBM SPSS Statistics 20, Armonk, NY, USA).

## 3. Results

This study was performed on CBCT images obtained from 100 patients (54 male and 46 female), with an average 42.12 ± 1.52 years of age (44.28 ± 2.25 and 39.54 ± 1.96, respectively), with no significant age difference between sexes (*p* = 0.122).

The evaluation of the first maxillary molars’ interradicular septum characteristics using sagittal CBCT slices showed no significant bilateral differences for all estimated parameters: the interradicular septum width at the A, B, C and D level; the interradicular septum height; and the angulation of furcation and the distance between the base of interradicular septum to the sinus floor (*p* = 0.482; 0.154; 0.095; 0.202; 0.225; 0.442 and 0.482, respectively). The analysis of sagittal CBCT slices of the second molar also confirmed no significant differences for contralateral sides of the interradicular septum for the same estimated parameters (*p* = 0.661; 0.138; 0.111; 0.135; 0.222; 0.422 and 0.521, respectively). The axial CBCT slices analysis again showed no significant side differences between the first maxillary molars’ interradicular septum perimeters at the levels A, B, C and D (*p* = 0.391; 0.789; 0.621 and 0.269, respectively), as well as for the surface area (*p* = 0.426; 0.857; 0.679 and 0.433, respectively). Almost the same results were obtained for the bilateral comparison of the parameters obtained on the second maxillary molars’ interradicular septum (*p* = 0.843; 0.057; 0.117; 0.127; and *p* = 0.717; 0.067; 0.162 and 0.211, respectively).

**Figure 3:**
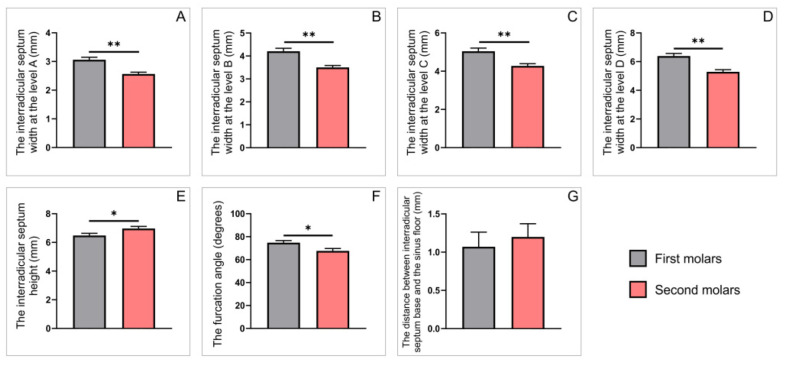
The parameters obtained on sagittal slices for all upper molars: (**A**–**D**) the interradicular septum width (in mm) at the levels A–D, respectively; (**E**) the interradicular septum height (in mm); (**F**) the interradicular furcation angle (in degrees); (**G**) the distance between interradicular septum base and the sinus floor (in mm). Values are expressed as the mean ± SEM. * Denotes a significant difference, *p* < 0.05; ** denotes a significant difference, *p* < 0.01.

The estimation of interradicular septum parameters between the first and second maxillary molars (a total number) obtained on sagittal CBCT slices is shown in Figure 3. The interradicular septum width of the first molars (Figure 3A–D) was significantly higher when compared to the second maxillary molars at all estimated levels (*p* < 0.01). In contrast, the interradicular septum height of the upper second molars was significantly higher when compared to the first molars (Figure 3E, *p* < 0.05). As shown in Figure 3F, the furcation angle of the first molars was significantly wider in comparison to the second molars (*p* < 0.05). However, the distance between the interradicular septum base to the sinus floor of the first and second molars showed no significant difference (Figure 3G).

**Figure 4:**
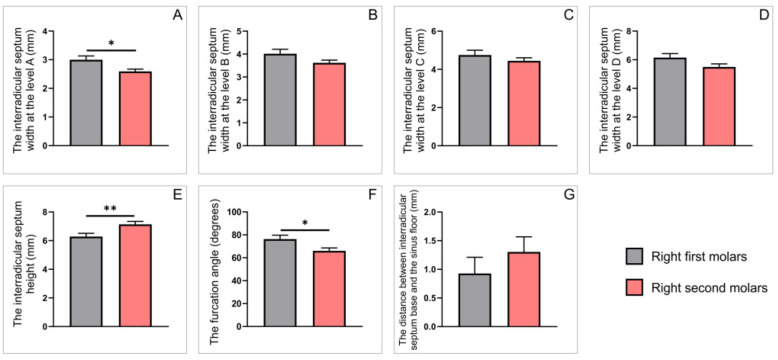
The parameters obtained on sagittal slices for upper molars on the right side: (**A**–**D**) the interradicular septum width (in mm) at the levels A–D, respectively; (**E**) the interradicular septum height (in mm); (**F**) the interradicular furcation angle (in degrees); (**G**) the distance between interradicular septum base and the sinus floor (in mm). Values are expressed as the mean ± SEM. * Denotes a significant difference, *p* < 0.05; ** denotes a significant difference, *p* < 0.01.

**Figure 5:**
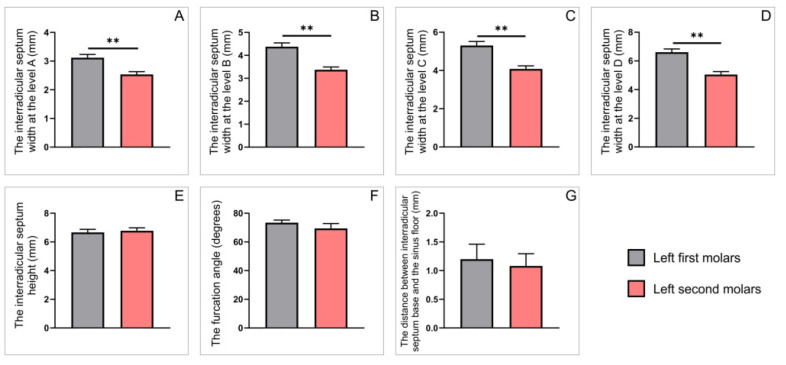
The parameters obtained on sagittal slices for upper molars on the left side: (**A**–**D**) the interradicular septum width (in mm) at the levels A–D, respectively; (**E**) the interradicular septum height (in mm); (**F**) the interradicular furcation angle (in degrees); (**G**) the distance between interradicular septum base and the sinus floor (in mm). Values are expressed as the mean ± SEM. ** Denotes a significant difference, *p* < 0.01.

The in-depth analyses that enlighten specific differences observed on either right (Figure 4) and left (Figure 5) side revealed the differences between the parameters for the first and the second maxillary molars (obtained on sagittal CBCT slices) were not identical. Namely, the significant differences between the first and second molars on the right side were confirmed by means of the interradicular septum width only at level A (Figure 4A, *p* < 0.05). The same parameter was significantly higher at all (A, B, C and D) levels on the left side (Figure 5A–D, *p* < 0.01). In contrast, the values for the interradicular septum height and angle furcation were significantly different only on the right side (Figure 4E,F; *p* < 0.01 and 0.05, respectively). Still, there was no difference between interradicular septum base to the sinus floor distances on either side (Figure 4G and Figure 5G).

**Figure 6:**
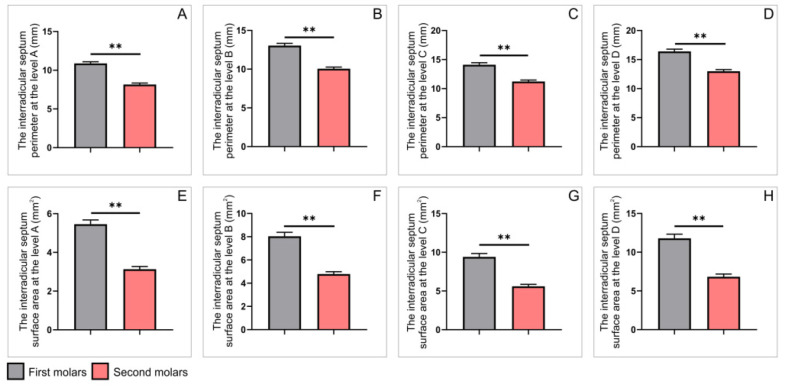
The parameters obtained on axial slices for all upper molars: (**A**–**D**) the interradicular septum perimeter (in mm) at the levels A–D, respectively; (**E**–**H**) the interradicular septum surface area (in mm^2^) at the levels A–D, respectively. Values are expressed as the mean ± SEM. ** Denotes a significant difference, *p* < 0.01.

As presented in Figure 6, the perimeters and surface areas of the interradicular septum obtained on axial CBCT slices were significantly influenced by maxillary molars’ position. The interradicular septum perimeters were significantly enhanced in the first when compared to the second molars at all estimated levels (Figure 6A–D, *p* < 0.01). Not surprisingly, a similar observation was achieved for the interradicular septum surface areas comparison between the first and the second upper molars. Again, the surface areas values for the first molars were significantly above the second maxillary molars at all estimated levels (Figure 6E–H, *p* < 0.01).

**Figure 7:**
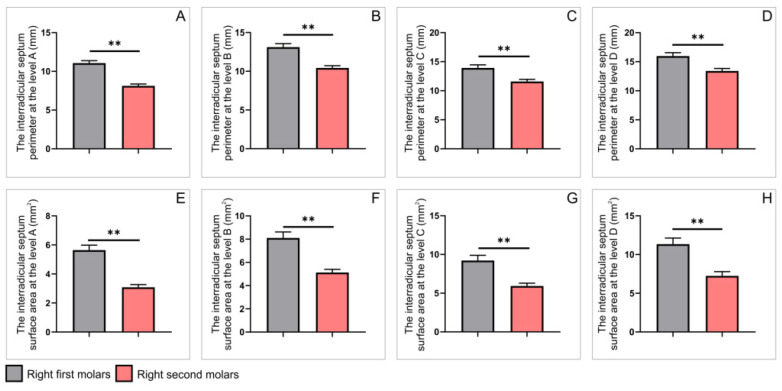
The parameters obtained on axial slices for upper molars on the right side: (**A**–**D**) the interradicular septum perimeter (in mm) at the levels A–D, respectively; (**E**–**H**) the interradicular septum surface area (in mm^2^) at the levels A–D, respectively. Values are expressed as the mean ± SEM. ** Denotes a significant difference, *p* < 0.01.

**Figure 8:**
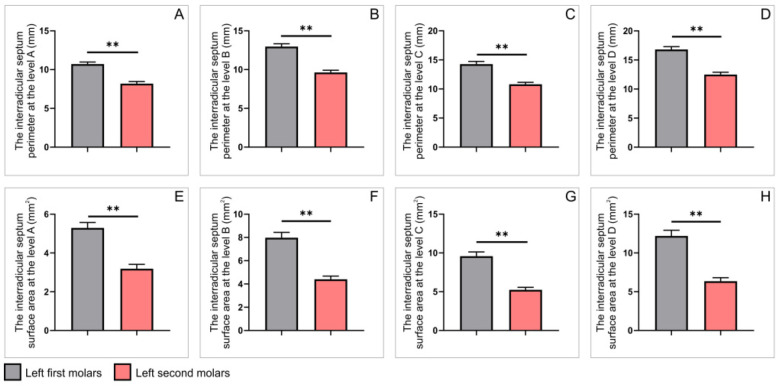
The parameters obtained on axial slices for upper molars on the left side: (**A**–**D**) the interradicular septum perimeter (in mm) at the levels A–D, respectively; (**E**–**H**) the interradicular septum surface area (in mm^2^) at the levels A–D, respectively. Values are expressed as the mean ± SEM. ** Denotes a significant difference, *p* < 0.01.

Interestingly, the analysis observed on axial CBCT slices, presented in Figure 7 and Figure 8, revealed that all estimated parameters showed the same type of statistical differences. Thus, both interradicular septum perimeters and surface areas were significantly higher in the right first upper molars when compared to ipsilateral second molars (Figure 7, *p* < 0.01). The very same observation was confirmed on the left side (Figure 8, *p* < 0.01).

**Figure 9:**
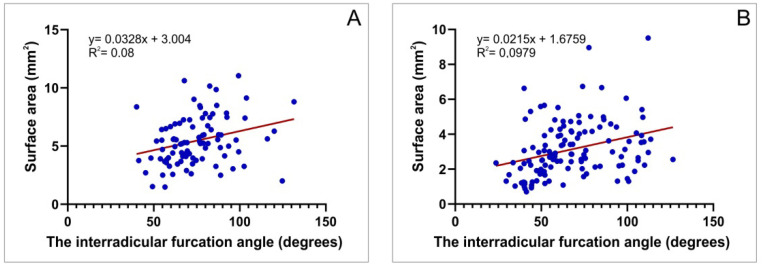
The correlation between the interradicular furcation angle and surface area at the level A: (**A**) the first maxillary molars and (**B**) the second maxillary molars.

In order to estimate the relationship between the furcation angle and surface area, we estimated that interconnection at the level A, as the most critical (narrow) point of clinical importance. The linear regression analyses revealed a significant positive correlation for both first and second maxillary molars (Figure 9A,B; R = 0.28 and 0.31; *p* = 0.0066 and 0.0004, respectively).

**Figure 10:**
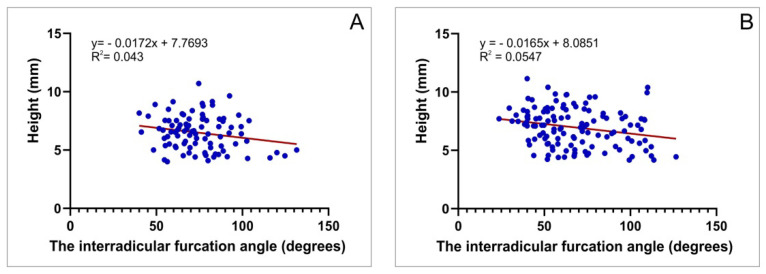
The correlation between the interradicular furcation angle and interradicular septum height: (**A**) the first maxillary molars and (**B**) the second maxillary molars.

Furthermore, we also estimated the relationship between the furcation angle and the height of interradicular septum. Again, we observed a significant correlation (only negative) for both the first and second upper molars (Figure 10A,B; R = 0.21 and 0.23; *p* = 0.0485 and 0.0089, respectively).

## 4. Discussion

The upper molars region is one of the most commonly reported for teeth loss [29,30,31]. According to current knowledge, a number of authors suggest an immediate implant placement as the best way for teeth restoration [5,32]. Furthermore, it has been confirmed that the post-extraction (immediate) socket has greater osteogenic potential in comparison to the mature (late) edentulous healed bone [33]. Thus, the rapid definition of some crucial morphometric characteristics of that maxillary region may be of significant importance for the final outcome of such interventions. The alveoli socket of the upper molar is a complex structure due to a higher prevalence of three separate roots [34]. From a prosthetic aspect, the interradicular septum is reported as an ideal anatomical structure for immediate implant placement in the area of the maxillary molar [35]. At the same time, it should be noticed that the interradicular septal bone could be absent or with insufficient dimensions to support the implant placement procedure [4]. Therefore, in order to allow for better insight into morphological variations of the interradicular septal bone, we analyzed the architecture of this specific region, intending to offer additional tools for clinicians in planning immediate implant placement.

Previous studies based on CBCT analysis described characteristic anatomical variations in the maxillary molars region that may be of clinical importance in relation to the implant placement procedures [13,14,15]. Additionally, CBCT ensures more detailed data when compared to more frequently used 2D radiographs [36]. Thus, it has been confirmed that CBCT has high accuracy in linear bone measurements [37], such as bone height, root proximity, and degree of furcation [25]. According to the enumerated advantages of CBCT, we used this imaging tool in the assessment of the interradicular septum bone morphology.

The results of our study demonstrated that no difference was noticed between the right and left (for both first and second upper molars) molars, neither on sagittal nor axial CBCT slices. The etiology of the observed phenomenon can be found in the study of Som and coworkers [38], who described symmetrical growth of maxillary sinuses.

However, the key point of our study was to estimate the differences between the interradicular septum bone morphological characteristics of the first and second maxillary molars using the CBCT technique. In order to allow for better insight into the interradicular septum properties, we performed linear measurements of its width at the four predefined levels. In that sense, the results obtained in this study showed that the first maxillary molars had a wider interradicular septum when compared to the second molars at all estimated levels. In contrast, the interradicular septum height values observed in the first molars were significantly below the height in the second maxillary molars. Unlike for the interradicular septum width, due to lack of literature data, the results considering the interradicular septum height obtained in this study have been previously described. However, this morphometric parameter was reported to be either enhanced [5] or lowered [25] in the first upper molars when compared to the second. According to Padhye and coworkers [24], the implant primary stability would be greatly compromised if the interradicular septum width is less than 3 mm. In this study we observed that the second molars had lower values of the interradicular septum width (at level A) when compared to the first molars (app. 2.6 vs. 3 mm). This should be taken into consideration for planning the immediate implant placement technique that may involve molar septum expansion with osseodensification in that position [21].

As Levin and colleagues [39] revealed that implant length also plays an essential role in its survival, Nunes and collaborators [22] recommended the minimum implant length of at least 10 mm, in order to achieve osseous anchorage (as a key factor for occlusal force resistance). This morphometric parameter imposes great clinical importance. Because the results of this study showed that interradicular septum height in the second molars was significantly above the values observed in the first upper molars (app. 7 vs. 6.5 mm), it seems necessary that certain surgical procedures (such as a sinus floor evaluation and vertical bone augmentation) may be differently applied in order to prevent failure of the implant therapy.

Furthermore, the distance between interradicular septum base to the maxillary sinus floor, which has not been evaluated before, also showed no significant differences according to the maxillary molar position, suggesting that this parameter may not be of significant clinical importance in different planning of immediate implant placement procedure for the first and second upper molars.

We also estimated the interradicular angle furcation, as a parameter that may significantly influence the planning of immediate implant placement procedures in the maxillary molar region. Our analyses showed that this parameter showed significantly higher values in the first molars (Figure 3F) when compared to the second molars, probably due to significant differences observed on the specific side (Figure 4F). Although this parameter has not been evaluated yet, its clinical implications (see below) may be considered as a potential checkpoint in the planning of immediate tooth placement procedures.

Using the axial CBCT slices, we evaluated the interradicular septum perimeter and surface area at A, B, C and D levels. The analyses strongly confirmed that both interradicular septum perimeter and surface area were significantly more pronounced in the first than in the second maxillary molars. This difference appeared congruently on either side. Although comparable only at the lower part of the interradicular septum (level A in this study), our results are in line with Agostinelli and colleagues [5], who reported that the interradicular septum of the first molars presented a dominant perimeter in comparison to the second molars. They also stated that the implant diameters in the range of 3.3 mm to 4.0 mm could not attain primary stabilization in larger alveoli, such as maxillary molars pockets, since the interradicular septum widths were insufficient to cover the full implant area, and that could be manifested as a remaining gap between interradicular septum and implant surface [5]. The results obtained in this study, which showed the significant prevalence of the interradicular septum surface area in the first molars in comparison to the second, further emphasize the importance of the previous classification by Smith and colleagues [19]. Altogether, this implies that in the cases where the gap is present between implants and the interradicular septum (more likely in the second upper molars, according to the results of this study) could be solved by grafting and/or using a wider implant [40].

We also estimated the perimeter and interradicular septum area of the first and second right molars using an axial cross-section. The first molar showed larger values of both (perimeter and the area) in comparison to the second molars. The same results were reported on the left side. This analysis confirms that immediate implant placement in the second molar area of both sides could require additional surgical procedures in order to achieve successful implant therapy.

Finally, the estimation of the interconnection between the interradicular furcation angle and surface area (performed at the critical point—level A) showed, as presented in Figure 9, a significant positive correlation, implying that the increase of the furcation angle leads to enhancement of interradicular septum width at the narrowest level for both upper molars. The clinical importance of this relationship may include the selection of dental implant characteristics according to the predefined interradicular furcation angle. Furthermore, the estimation of the correlation between the furcation angle and interradicular septum height revealed a significant negative correlation for both first and second maxillary molars, leading to the conclusion that the preliminary defining of the furcation angle should be involved as an additional criterion for choosing the implant length.

## 5. Conclusions

The results of this study obtained using CBCT images may recommend this technique as a useful tool in predefining the circumstances that can allow for substantially better planning of immediate implant placement procedures in the region of maxillary molars. Additionally, it could be noticed that, in most cases, the interradicular septum of the first maxillary molars allowed for wider implant (in diameter) placements, while its characteristics in the second maxillary molars could be directed to the selection of the enhanced length (instead of the width) implants in order to achieve primary stability.

## Data Availability

Data are available upon request from the authors.
